# Predictors of low dental service utilization among school children in Mekelle, Northern Ethiopia: a cross-sectional study

**DOI:** 10.1186/s12903-023-02740-6

**Published:** 2023-01-25

**Authors:** Hayat Maeruf Mohammed, Mihret-ab Mehari, Akeza Awealom Asgedom

**Affiliations:** 1 College of Medicine and Health Sciences, Adigrat University, P.O. Box 50, Adigrat, Ethiopia; 2 College of Health Sciences, Mekelle University, Mekelle, Ethiopia; 3 Department of Environmental Health and Behavioral Sciences, School of Public Health, College of Health Sciences, Mekelle University, Mekelle, Ethiopia

**Keywords:** Dental service utilization, Ethiopia, Mekelle, School children, Tigray

## Abstract

**Background:**

Dental service utilization is important for maintaining and enhancing children’s oral health status. However, there is paucity of information regarding dental service utilization and factors affecting it among school aged children in Ethiopia.

**Objectives:**

The purpose of this study was to determine the dental service utilization and associated factors among school aged children (6–15) years in Mekelle city, Northern Ethiopia.

**Methods:**

A school-based cross-sectional study was conducted in Mekelle city of Northern Ethiopia from January 2016 to June 2016. A multi-stage sampling method was used to select 405 school children. A modified World Health Organization oral health assessment form for children was used to collect data. Univariate and multivariable logistic regressions with 95% CI were used to test the association between past-year dental service utilization, as an outcome variable, and parental socio-demographic, and child characteristics as independent variables.

**Results:**

A total of 398 school children participated in the study. The overall dental service utilization among these children was (10.6%), 95% Confidence Interval (CI) (7.5%, 13.6%). In multivariable logistic regression analysis, maternal educational status (illiterate versus college and above (adjusted odds ratio (AOR) 0.13, 95% CI 0.01, 0.93)), higher monthly income (AOR 11.69, 95% CI 1.19, 114.61)), and having dental pain (AOR 50.8, 95% CI 17.8, 145.17)) were significantly associated with past year dental service utilization.

**Conclusion:**

Our findings showed that a small proportion of the study population visited a dentist in the past year. Maternal educational status, monthly income, and dental pain were associated with past year dental service utilization. Oral health education programs focusing on dental service utilization targeting school children are crucial.

## Background

Oral health is important for overall health, well-being, and quality of life at all ages [1]. Good oral health is key to eating, speaking, and socializing without pain [2]. It is also essential for children’s physical, psychological, cognitive, social, and academic development [3]. The best way to maintain oral health status is by making regular dental visits to health facilities to obtain preventive, diagnosis, and proper treatment services [2]. The American Academy of Pediatric Dentistry recommends that the first dental visit, followed by regular dental visits every six months, should begin at age one [1]. Dental health service utilization can be measured by the number of visits to health facilities per year [2].

Due to the increasing sugar consumption, insufficient fluoride exposure, and limited access to oral health care services, oral diseases are increasing in many African countries [4, 5]. Dental caries, acute necrotizing ulcerative gingivitis (ANUG), and cancrum oris (NOMA) are common among school children and left untreated in Africa [6]. Worldwide, disability-adjusted life-years (DALYs) due to oral conditions have increased by 20.8% in the past 20 years (1990–2010), and the largest increase was observed in Sub-Saharan Africa, where Ethiopia is also located [7]. In Africa, dental caries prevalence among school children was about 60–80% as of 2015 [8]. A few studies conducted in Ethiopia showed a prevalence of dental caries ranging from 20 to 75% [9–11]. Unpublished survey conducted in Mekelle city also showed a prevalence of 40% dental caries among school children [12].

The availability and access to oral health care in almost all African countries is very limited due to unequal distribution of health care professionals, inadequate facilities, lack of dental insurance, and lack of budget as most priority is given to the major concerns i.e. communicable diseases [13, 14]. In addition, the use of dental services is mainly for symptomatization [14].

There is paucity of evidence regarding dental service utilization in Africa. Few studies have shown that the use of dental services by African children is lower compared to other non-African countries. For instance, a report from Nigeria showed that school children’s use of dental services was reported to be only 14% [2, 15] which is lower as compared to the evidence from America with a dental service utilization ranging from 66 to 76% [1, 16, 17], Spain 65% [18], and in Saudi-Arabia 61.7% [19].

The use of dental service has a broad range of predicting factors, ranging from child’s gender, age, ethnicity, residence, and insurance status, presence of dental pain to family factors including income, educational status, and occupation [19, 20].

There is little attention given to access and use of oral health services in Ethiopia, where there is a high prevalence of communicable diseases, including HIV, TB, and malaria [9]. In addition Ethiopia’s population-to-dentist ratio (> 100 million total population) was 1: 1 million [21] leading to inadequate provision of dental services for children and adults. Furthermore, there is no school and community based oral health programs in Ethiopia. On top of this, there is no dental insurance covering the associated costs for dental care targeting children and other segments of the population. As a result, clients are expected to get dental services through out of pocket payments which is challenging to afford for most of the community. According to Tigray regional health bureau 2022 report, there are around thirty private medium clinics, two public general hospitals, and one public referral hospital giving dental care services in Mekelle.

It is estimated that 71% of the Ethiopian population is affected by some form of oral disease. Nevertheless, awareness and dental service use is very limited [22]. There is lack of data showing the factors affecting the use of dental services in Ethiopia in general and Tigray in particular. To the best of our knowledge, there is no study regarding dental service utilization by children in Mekelle and the country as a whole. Therefore, the present study was aimed at determining dental service utilization and their associated factors among school-aged children in Mekelle, Ethiopia.

## Methods

### Study area and population

This school-based cross-sectional study was conducted in Mekelle city of Northern Ethiopia from January 2016 through June 2016 to determine dental health service utilization and associated factors among school children. The city is located 783 km away from the capital city, Addis Ababa. The populations of school children aged (6–15 years old) were 69,208. The city has 67 urban primary schools within seven sub-cities where 44 were public and 23 were private schools. School children from both public and private schools participated in the study.

### Sampling method

The sample size 405 was calculated using a single population proportion formula based on the assumption that the level of dental service utilization of 14.7% taken from Nigerian study [2], with an assumption of 95% confidence level, 5% degree of precision, a design effect of 2, and 5% non-response rate giving a final sample size of 405. A multistage stratified sampling technique was used to select study subjects. The first step involved a random selection of four sub-cities from seven sub-cities. The second step involved a selection of ten primary schools by proportional allocation from selected sub-cities. The third step involved proportional allocation to each school and grade level (1st up to 8th ). Finally, after taking representative classes from each grade level, study subjects were selected proportionally based on their sex using systematic random taking their roster as the sampling frame.

### Data collection methods

The parents of the primary school children were invited to complete a self-administered questionnaire written in *Tigrigna* (the local language) at school. Parents, who were unable to read and write, were interviewed using a structured instrument with the same items as the self-administered questionnaire.

A pretest was done in an area outside of the actual study regarding the wording, meaning, and feasibility of our data collection tool. We used a modified standardized questionnaire from the 2013 World Health Organization Oral Health Assessment form for children. The questionnaire consisted of a detailed briefing on the child’s age, gender, grade level, dental health status (perceived dental pain), and previous dental visit including its reason and frequency. In addition, the socio-demographic characteristics of the children’s parents such as self-reported monthly income and educational status were collected. Income level was categorized based on the 1 USD daily income (30 USD monthly income (there are 30 days in a month) ~ 1000 Ethiopian Birr) as cut point for poverty. The 1000 was used to regroup the income level further.

Before the study, three dental hygienists were given training for one day by the principal investigator. The training focused on the purpose of the study, how to conduct face to face interviews as well as fill the questionnaire. Later, the questionnaire data were entered twice and inconsistencies were removed by cross-checking the questionnaire.

### Measurement of the outcome variable

The outcome variable was dichotomized to indicate if the child had a past year dental visit or not.

### Data analysis

Data collected from interviews and self-administered questionnaires were used for analysis. All the statistical analysis for this study was conducted with SPSS version 20. Descriptive statistics were carried out, followed by univariate analysis using cross-tabulations to obtain column percentages of each variable in relation to the past year dental visit, and Pearson’s chi-square statistical test. To determine the factors associated with past year dental service utilization, univariate and multivariable logistic regression with the existence of past year dental service utilization as the outcome variable (No = 0, Yes = 1), and child factors (sex, age, grade level, perceived dental health status (pain)), and family factors including educational status and a monthly income as the independent variables was performed. Before performing the logistic regression, we tested the presence of multicollinearity between independent variables and no multicollinearity was detected.

To determine the association of each variable in the model, we first performed univariate analysis and set a p-value < 0.3 as a threshold to filter variables that should be included in the multivariable analysis. All variables that passed the threshold were included in the multivariable analysis. The Goodness-of-fit of the model was checked by the Hosmer-Lemeshow test. The association of individual variables with the presence or absence of past year dental service utilization was stated in (adjusted) odds ratio with its corresponding 95% confidence interval (CI). In the multivariable analyses, significance was set at p-value < 0.05.

## Result

### Socio-demographic characteristics of school children

A total of 398 primary school children participated in the study giving a response rate of 98.2%. The mean (SD) age of study participants was 10.41 (2.44) years old. Female school children accounted for half (50%) of the study participants. More than half, 204 (51.3%) of the study participants attended grade one up to four. Close to one fourth (27.6%) of the children were living in households with monthly incomes of less than ETB 1000 (~ USD30). A large number of school children 87 (21.8%) had dental pain in the last 12 months (Table 1).

**Table 1:** Socio-demographic and dental characteristics of school children in Mekelle city (n = 398)

Variable	Category	Dental service utilization		Total N	P-value
		Yes N (%)	No N (%)		
Age	6–10	17 (40.5)	187 (52.5)	204	0.139
	11–15	25 (59.5)	169 (47.5)	194	
Sex	Male	24 (57.1)	175 (49.2)	199	0.328
	Female	18 (42.9)	181 (50.8)	199	
Grade level	1–4	18 (42.9)	186 (52.2)	204	0.250
	5–8	24 (57.1)	170 (47.8)	194	
Mother’s education	Illiterate	2 (4.8)	89 (25)	91	0.002
	Read and write only	3 (7.1)	14 (3.9)	17	
	1–8	14 (33.3)	125 (35.1)	139	
	9–12	15 (35.7)	58 (16.3)	73	
	College and above	8 (19)	70 (19.7)	78	
Father’s education	Illiterate	2 (4.8)	45 (12.6)	47	0.262
	Read and write only	1 (2.4)	22 (6.2)	23	
	1–8	15 (35.7)	116 (32.6)	131	
	9–12	6 (14.3)	69 (19.4)	75	
	College and above	18 (42.9)	104 (29.2)	122	
Monthly household income in ETB ^ᾩ^	≤ 1000	5 (11.9)	105 (29.5)	110	0.133
	1001–2000	11 (26.2)	54 (15.2)	65	
	2001–3000	7 (16.7)	55 (15.4)	62	
	3001–4000	2 (4.8)	18 (5.1)	20	
	4001–5000	4 (9.5)	28 (7.9)	32	
	> 5000	13 (31)	96 (27)	109	
Dental pain experience in the past 12 months	Have dental pain	35 (83.3)	52 (14.6)	87	< 0.001
	No dental pain	7 (16.7)	304 (85.4)	311	

ETB-Ethiopian Birr; ^ᾩ^ 1 USD = 34 ETB

### Dental service utilization

The overall level of dental service utilization in the past 12 months by school children was 10.6%, 95% CI (7.5%, 13.6%). A high fraction, 356 (89.4%) of study participants had never seen a dentist. Majority of (57.1%) of the 42 children who had prior dental visits were boys, while the remaining children were girls. Of the children who had never had dental visits, 181(50.8%) were girls, while the remaining 175 (49.2%) were boys. Different reasons were given for dental service utilization; dental pain 34 (80.9%), 4 (9.5%) dental checkups followed by other dental procedures (Orthodontics and braces) 4 (9.5%). Of the 42 children who have had previous dental visits 29 (69%) visited once, 9(21.4%) visited twice, while the reaming 4(9.5%) visited three times and above.

### Factors associated with dental service utilization in the past year

In univariate analysis, sex of the child had P-value > 0.3 and was not included in the multivariable analysis. Multivariable regression identified factors significantly associated with children’s past-year dental service utilization (Table 2). Children with illiterate mothers were 87% less likely to have dental service utilization in the past 12 months (Adjusted Odds Ratio (AOR) = 0.13, 95% Confidence Interval (CI) = 0.01–0.93)) compared to whose mothers had attended college and above. Children who lived in households with a higher monthly income of (3000–4000 ETB) had higher odds (AOR = 11.69, CI = 1.19–114.6)) to have a past year dental visit than those children from households with a monthly income of less than 1000 ETB. The odds of past-year dental service utilization were fifty times higher for children with dental pain than those without (AOR = 50.86, CI = 17.82–145.17)) (Table 2).

**Table 2:** Factors associated with dental service utilization among school children in Mekelle city, 2016 (n = 398)

Variables	Category	COR (95% CI)	AOR (95% CI)	p value
Age	6–10	0.61 (0.32–1.17)	0.20 (0.03–1.06)	0.060
	11–15	1	1	
Grade level	1–4	1.45 (0.76–2.78)	3.33 (0.63–17.42)	0.153
	5–8	1	1	
Mother’s education	Illiterate	0.19 (0.40–0.95)	0.13 (0.01–0.93)*	0.043*
	Read and write only	1.87 (0.44–7.95)	2.59 (0.34–19.72)	0.358
	1–8	0.98 (0.39–2.45)	0.56 (0.14–2.26)	0.422
	9–12	2.26 (0.89–5.71)	0.93 (0.25–3.47)	0.918
	College and above	1	1	
Father’s education	Illiterate	0.25 (0.05–1.15)	0.16 (0.02–1.24)	0.080
	Read and write only	0.26 (0.03–2.07)	0.08 (0.06–1.19)	0.067
	1–8	0.74 (0.35–1.55)	0.79 (0.23–2.71)	0.715
	9–12	0.50 (0.19–1.32)	0.27 (0.69–1.11)	0.071
	College and above	1	1	
Monthly household income in ETB ^*ᾩ*^	≤ 1000	1	1	
	1001–2000	4.27 (1.41–12.94)	4.04 (0.94–17.3)	0.060
	2001–3000	2.67 (0.81–8.81)	2.88 (0.60–13.70)	0.183
	3001–4000	2.33 (0.42–12.95)	11.69 (1.19-114.61)	0.035*
	4001–5000	3.00 (0.75–11.91)	1.55 (0.23–10.1)	0.644
	> 5000	2.84 (0.97–8.27)	2.19 (0.49–9.65)	0.300
Dental pain experience in the past 12 months	Have dental pain	29.23 (12.33–69.29)	50.86 (17.82-145.17)	< 0.001*
	No dental pain	1	1	

*COR* Crude odds ratio; *AOR* Adjusted odds ratio; *CI* Confidence interval

*Significant at p < 0.05

^*ᾩ*^Statistical significance was taken at p value ≤ 0.05

## Discussion

The study determined the level of dental service utilization for school children [6–15] years old, showing that only one child from ten children (10.6%) had dental visits in the past year. Our results showed that the level of dental service utilization was very low among school children attending both public and private schools in Mekelle, Ethiopia. The finding is similar to the studies done among school children in Nigeria [2]. However, the present finding is lower than the studies from the United States of America with dental service utilization ranging from 66 to 76% [1, 16, 17], Australia (77%) [23], and Canada (54.8%) [24]. This difference could be explained due to the country’s socio-economic disparities, unavailability of health care services and health seeking behaviors of the community.

This study identified an association between maternal educational level and dental service utilization which is similar with the findings in America [1, 16]. Another systematic review also indicate that people with lower educational status have lower utilization of dental services as compared to those with higher educational status [20]. This could be related to the difference in their knowledge regarding the benefits of dental visits and their attitude in utilizing it.

Family income was also associated with dental service utilization in the present study which is in agreement with the studies done in Nigeria [2], Saudi Arabia [20] and America [1]. As there is no insurance for dental health and the service is out of pocket payment in Ethiopia, those with low family income cannot afford the associated costs of dental services.

In this study, nearly 34 (81%) of dental service utilization was due to dental pain. This shows that the chances of a healthy dental checkup are low. Accordingly, children with dental pain had higher odds of using dental service which is consistent with the results of other studies [1, 19]. This could be linked to the associated pain and discomfort that might force the family to take their children to the dentist for the required dental service.

In general, dental pain, family income, and maternal educational levels were significantly associated with past year dental service utilization with wide confidence intervals. The wide confidence interval may arise due to smaller sample size considered in our study. This smaller sample size could have its own drawbacks such as introducing a sampling error. Therefore, the interpretation of the finding should consider the context of the study and a future study considering large sample size is recommended.

This cross-sectional study showed that the overall use of dental services in Mekelle city is very low. The implication of this finding is to incorporate oral health education into the school curriculum as there is no oral health school program in Ethiopia in general and Tigray in particular. The outcome of this study can be used by administrators of schools, non-governmental organizations, and government agencies/departments to introduce oral health programs in school activities. The standardized data collection tool and cross sectional design are appropriate for prevalence studies and may support the validity of the results. However, this study had some limitations. The study did not explore the knowledge, attitude, and reasons for non-use of dental service. There could be also other associated factors that are not addressed in the present study such as medical condition of the child. In addition, the study did not assess the oral health status of school children by documenting the decayed, missed, and filled (dmf/DMF) teeth of school children which may have an impact in dental service utilization. This could be a possible research topic for the future.

Future studies considering the current limitations are recommended to fill the gap, and this could help overcome some of the barriers associated with the use of dental services for children.

## Conclusions

The present finding showed that a small proportion of the population has visited a dentist in the past year. Maternal educational status, monthly income, and dental pain were associated with past year dental service utilization. Oral health education and programs focused on the use of dental services are essential to promote oral health in schools.

## Data Availability

The dataset used during the current study are available from the corresponding author on reasonable request.
